# Equifinality and multifinality in developmental paths to a hostile mindset: A longitudinal study of mother–child and father–child dyads from toddler to early school age

**DOI:** 10.1017/S0954579426101333

**Published:** 2026-03-27

**Authors:** Grazyna Kochanska, Juyoung Kim, Haley Michelle Herbert

**Affiliations:** Department of Psychological and Brain Sciences, The University of Iowa, Iowa City, USA

**Keywords:** Hostile mindset, Parenting, Theory of mind, Regulation, Children’s representations, Longitudinal studies

## Abstract

Studies persuasively show that parental power assertion contributes to children’s hostile (defensive) mindsets, but most examined severe forms of control (abuse, harsh punishment) and aggressive children. Less is known about processes linking power assertion with children’s hostile mindsets in typical, low-risk families. Further, specific mechanisms accounting for associations between parenting and hostile mindsets are unclear; children’s theory of mind (ToM) and regulation have been suggested, implying equifinality in developmental cascades. Finally, factors that moderate impact of parenting on children’s hostile mindsets, implying multifinality, are unclear. In a study of 200 mothers, fathers, and children, we proposed that links between parental power assertion and children’s hostile mindsets are (a) accounted for by two parallel mediators – children’s poor ToM and poor regulation, and (b) moderated by their representations of parents. We expected links between power assertion and hostile mindset to be significant for children with negative representations, but defused, or absent, for children with positive representations. Parental power assertion was assessed at toddler and preschool age, ToM and regulation at preschool age, and hostile mindsets and representations of parents at early school age. We supported both mediated paths for mother–child dyads, mediation via child regulation for father–child dyads, and moderation for both.

Over the last several decades, Dodge and colleagues’ research on children’s hostile social-information processing has inspired one of the most theoretically influential and empirically supported perspectives on the development of aggression and conduct problems worldwide (e.g., Crick & Dodge, 1994; Dodge, 1980, 2024; Lansford et al., 2010). Voluminous research, whose review is beyond the scope of this article, has persuasively documented that aggressive children exhibit hostile biases when processing ambiguous social information. Those biases are evident at multiple processing steps, from being hypervigilant toward potentially hostile cues, to attributing hostile intent to others, to prioritizing hostile action plans. Recently, Dodge and colleagues (Dodge, 2024; Dodge et al., 2022) proposed an important shift from this earlier approach, which focused on hostile biases in the separate steps children deploy as they process ambiguous social information, to a more trait-like, general latent construct of “hostile (or defensive) mindset”, conceptualized as a broadly negative worldview that guides their social and emotional functioning.

Since the seminal paper on the key role of abusive, harsh parenting in the origins of children’s hostile mindsets (Dodge et al., 1990), robust empirical evidence has supported the role of harsh control (e.g., child maltreatment, corporal punishment, yelling, and physical and verbal threats) as a risk factor for hostile biases in social information processing and hostile mindset in peer contexts (Dodge, 2006; Nelson & Coyne, 2009; Weiss et al., 1992). That research has often focused on families and children oversampled for harsh, even abusive discipline, and for children’s externalizing behaviors and conduct problems.

Of note, researchers have also studied other negative parenting aspects as associated with children’s hostile mindsets, such as rejection, unresponsiveness, or intrusiveness (e.g., Bodecka-Zych et al., 2025; Healy et al., 2015), as well as positive dimensions, such as affective mutuality or insightfulness (Czik et al., 2025; McElwain et al., 2008). For the purpose of the present review, however, we focus on the parental dimension of control, and specifically power assertion, harshness, punitiveness, sometimes referred to as authoritarian discipline, either observed or reported. It is important to keep in mind that in various studies, the constructs of power assertion have varied widely in types and intensity of behaviors they denoted – from mild, firm, or strict verbal control, warnings, rebukes, or minor threats, occasional light spanking, restricting activities, and non-physical punishments, part of typical parenting in virtually all families – to severe or abusive child maltreatment. This complex nature of the concept of power assertion is well known (Hoffman, 1983; Larzelere et al., 2024; Weiss et al., 1992).

Generally, the extant evidence has consistently demonstrated that parental increased reliance on power-assertive strategies is associated with increased risk of children forming hostile mindsets. Hostile mindsets have been typically assessed as children’s responses to sequential questions targeting their social-information processing, elicited by vignettes depicting acts of provocation or rejection by peers whose intentions are portrayed as ambiguous.

As examples, Runions and Keating (2007), using data from the large NICHD study of early child care, reported that mothers’ self-endorsed authoritarian attitudes, assessed in infancy, predicted children’s hostile attributions of intent and aggressive response planning in measures of their mindsets in first grade, although observed negative parenting across the first three years was unrelated to those measures. Ziv and Arbel (2020) reported similar findings, such that mothers’ authoritarian parenting and perception of conflict with their children were related to children’s tendency to generate incompetent social responses, and further mediated the link between mothers’ and children’s hostile attributional biases. Ziv et al. (2016) reported that observed maternal negative control in interactions with their 5-year-old children predicted some aspects of children’s hostile social-information processing (maladaptive response evaluation). Gomez et al. (2001) found that children who perceived their mothers’ discipline negatively had higher scores in several steps of hostile information processing measures a year later. Weiss et al. (1992) found that parent-reported harsh discipline strategies were associated with children’s greater hostile processing biases in two large cohorts of preschool children; in those samples, Dodge et al. (1995) found that experiences of physical abuse, reported by the parents, were positively associated with children’s multiple hostile processing biases. Galán et al. (2017) found that mothers’ observed punitiveness during interactions with their toddler sons predicted children’s more hostile mindsets at age 10, although the findings were mostly for African American families.

The associations between harsh or power-assertive parenting and children’s hostile mindsets have been typically explained in terms of the consequences of early relational experiences on the process of forming one’s internal models of the social world. Early experiences of threatening, harmful, or harshly punitive discipline impact children’s worldviews and expectations of others, which generalize and naturally lead to vigilance toward others’ potentially threatening actions, attributions of hostile intent to others, and reliance on defensive or aggressive responses (Dodge, 2024; Dodge et al., 1990). Such a perspective closely dovetails and is synergistic with attachment-informed models, in which children’s hostile or benign worldviews are seen as formed in the context of early parent–child relationships and ultimately become broader interpretive filters, generalizing from early experience (Bowlby, 1969/1982; Bretherton & Munholland, 2008; Carlson et al., 2004; Dykas & Cassidy, 2011; Dykas et al., 2011; L. A. Sroufe et al., 2010; Thompson, 2021). Indeed, Czik et al. (2025) explicitly applied the attachment theory lens to research on origins of children’s hostile biases in social-information processing.

Such a view is certainly conceptually compelling and supported by empirical evidence, and yet, that research opens several research questions that invite a fuller exploration. One, what specific mechanisms account for the effects of power-assertive control on children’s hostile mindsets? Such mediators have not been extensively studied. As the first aim of our work, we test *an equifinality hypothesis, which proposes that early parental power-assertive control leads to children’s hostile mindsets via two paths: a diminished ability to understand others’ perspectives, or theory of mind (ToM) skills, and poor regulation capacities.* Both ToM and regulation develop within the social context of the parent–child relationship during early years, and they are crucial skills for properly interpreting and reacting to social situations (e.g., Hughes & Leekam, 2004; Olson & Lunkenheimer, 2009).

Of note, the link between power-assertive parenting and children’s antisocial, aggressive outcomes, although common, is not universal. Multiple studies have indicated that those relations are more nuanced and can be conditioned on other factors. For example, although detrimental in White families, power-assertive parenting may not have negative consequences in Asian or African American families (Chao, 1994; Deater-Deckard & Dodge, 1997; Deater-Deckard et al., 1996; Lansford et al., 2004; Stacks et al., 2009).

This body of research has inspired our second question: What factors can moderate the effects of negative control on children’s hostile mindsets? Very little research has examined this question. As the second aim, we test *a multifinality hypothesis, which proposes that children’s representations of the parent as responsive and reliably providing comfort and effective help in times of stress can buffer the negative effects of parental power-assertive control.* We draw on recent findings that have robustly supported such moderation effects in several longitudinal studies that considered a range of socialization outcomes (Herbert et al., 2026; Kochanska & An, 2024a, 2024b; Kochanska et al., 2019). The comprehensive model, inspired by a developmental psychopathology framework (Cicchetti & Rogosch, 1996) and integrating equifinality and multifinality, is depicted in Figure 1.

**Figure 1.**
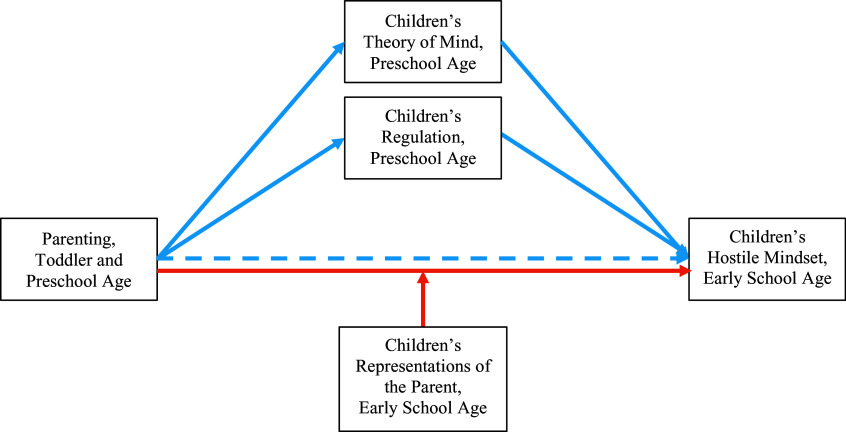
Proposed model of equifinality and multifinality in developmental paths from parenting to children’s hostile mindset. Blue lines represent paths related to equifinality (i.e., parallel mediation via theory of mind and regulation), and orange lines represent paths related to multifinality (i.e., moderation by children’s representations of the parent).

## The first aim: equifinality in paths from parenting to hostile mindset, with the child’s ToM and regulation as parallel mediators

### Children’s ToM as a mediator of links between parenting and hostile mindset

***Parenting and ToM.*** ToM refers to the ability to understand others’ perspectives and behaviors by attributing mental states (Devine & Hughes, 2018). Literature linking broadly conceived qualities of parenting with children’s ToM skills is vast, beyond the scope of this article. Multiple studies and reviews have linked various aspects of negative parenting, such as negative affect and harsh control, including maltreatment, to children’s diminished ToM and other aspects of social cognition (Hughes et al., 1999; Olson et al., 2011; see also reviews by Luke & Banerjee, 2013; Pavarini et al., 2013).

***ToM and Hostile Mindset.*** Although surprisingly limited, there is also evidence of associations between children’s ToM and their hostile mindsets. Killen et al. (2011), in two studies of children between ages 3.5 and 7.5, found that children who had failed to pass ToM tasks were more likely to attribute hostile intentions and to find harsh punishment acceptable in response to vignettes depicting “an incidental transgressor”. Studying young adults, Koo et al. (2022) found that those with lower ToM skills were more likely to show hostile biases in their interpretations of ambiguous vignettes. However, not all studies have supported associations between ToM and hostile mindsets (van Dijk et al., 2018).

### Children’s regulation as a mediator of links between parenting and hostile mindset

***Parenting and Children’s Regulation.*** Regulation is an extremely broad umbrella term that encompasses multiple phenomena and constructs such as self-control, delay of gratification, inhibitory control, effortful control, executive functions, and many others (Cole et al., 2019; Eisenberg et al., 2024; Gagne, 2017; Kopp, 1982; Rothbart & Bates, 2006), at times referred to as the “self-regulation universe” (Nigg, 2017). The literature on the role of parenting in the development of regulation is equally massive (Boldt et al., 2020; Bridgett et al., 2015; Corapci et al., 2025; Eisenberg et al., 2024; Kochanska et al., 2000; Thompson, 2006). Although a review is beyond the scope of this article, robust evidence supports a detrimental role of power-assertive parental control on regulation, even in community families, in which control is rarely harsh (Čepukienė & Janulevičė, 2025; Kochanska & Knaack, 2003), although multifinality in those effects has also been documented (Chang et al., 2011; J. Kim & Kochanska, 2026a; S. Kim & Kochanska, 2026).

***Regulation and Children’s Hostile Mindset.*** As mentioned, regulation can encompass a broad variety of phenomena, and many of its aspects have been studied in relation to children’s hostile information processing, generally indicating that better regulation was associated with less hostile and more adaptive mindsets. Most pertinent studies have examined emotion regulation in connection with hostile mindsets, following Lemerise and Arsenio’s (2000) proposal that emotion (dys)regulation influenced all steps of children’s social-information processing outlined by Crick and Dodge (1994). In their review of pertinent literature, Smeijers et al. (2020) identified four studies that confirmed an association between emotion dysregulation and hostile social-information processing (Calvete & Orue, 2012; Gagnon et al., 2015; Helmsen et al., 2012; Orobio de Castro et al., 2005). Colton et al. (2023) found positive links between college students’ self-reported emotion dysregulation and their hostile attributional biases.

Some studies have examined executive function skills as measures of regulation. Caporaso et al. (2021) found that preschool children with better pertinent skills (e.g., working memory, cognitive flexibility) engaged in more adaptive, or less hostile, social-information processing steps.

### Parenting, ToM, and regulation as predictors of hostile mindset

Only a few studies have included several factors – ToM, regulation, and parenting – as predictors of children’s hostile mindset. In a group of children oversampled for conduct problems, Choe et al. (2013) examined their ToM and regulation (using effortful control tasks) at ages 3.5 and 5–6, and hostile mindsets at age 5–6. Better ToM skills robustly predicted lower levels of hostile mindsets. Better regulation was also robustly related to lower hostile mindsets in correlational analyses (although not in complex total models that included several other predictors). Regulation also moderated the effects of ToM on hostile mindsets. However, no parenting measures were considered.

One study, predicting hostile mindsets from ToM and latent profile membership of executive function, revealed complex longitudinal relations depending on the type of hostile mindsets and ToM (i.e., cognitive and affective), executive function profile membership, and child gender (Brandt et al., 2025). This study, however, did not measure parenting and included older participants (early adolescence at the first time point and late adolescence to emerging adulthood at the second time point).

Using the same sample as Choe et al. (2013), Lee et al. (2019) examined mother-reported use of harsh discipline, and children’s ToM, regulation, and hostile mindset at age 6. There were significant negative correlations between ToM and hostile mindset and between regulation and hostile mindsets, and positive correlations between maternal reports of harsh control and hostile mindsets. Those relations, however, were not significant in the overall regression with multiple other constructs, including maternal attributional styles regarding child behavior.

Although these studies were very valuable in delineating associations among parenting, children’s ToM, regulation, and hostile mindsets, they did not specifically test formal mediational paths from parenting to ToM and regulation to hostile mindsets. Further, the parenting measures were based on mothers’ reports.

## The second aim: multifinality in paths from parenting to hostile mindset, with the child’s representation of the parent as a moderator

Multifinality – one of the key tenets of developmental psychopathology – refers to the assumption that a causal factor may lead to different outcomes and trigger varying developmental paths, depending on other factors or their combinations (Cicchetti & Rogosch, 1996). To our knowledge, however, few, if any, studies have applied the multifinality perspective to examine associations between negative parenting and children’s hostile mindsets.

Although detrimental effects of parental physical, hostile, forceful, harshly power-assertive, and abusive control are well recognized (e.g., Dodge et al., 1990; Gershoff, 2002), there is much less consensus on milder forms of power assertion, non-abusive discipline, routinely practiced by parents in typical, low-risk families during interactions with their young children (Larzelere et al., 2018; Lin et al., 2020; Ritchie, 1999). Such power assertion may include direct, strong, firm, insistent commands or prohibitions, issued in a “means-business”, decisive tone or in a raised voice, warnings about potential consequences for noncompliance, taking a toy away from a child, use of mild physical pressure to force child attention when insisting on compliance, stopping the child physically from a prohibited behavior, or mild spanking on rare occasions.

As reviewed earlier, research has persuasively indicated that effects of such common forms of power assertion can be substantially moderated by other factors, such as culture or ethnicity. As well, qualities of the parent–child relationship, such as security of attachment, have been shown to moderate effects of power-assertive control (see Bendel-Stenzel et al., 2023; S. Kim & Kochanska, 2026; Kochanska & An, 2024b; Kochanska et al., 2019; Kochanska & S. Kim, 2012 for reviews). Research has also supported the moderating role of children’s perception of parental power assertion; in an international large study, effects of harsh discipline were more adverse when children perceived it as being nonnormative than when they perceived it as being normative (Gershoff et al., 2010; Lansford et al., 2010). Most recently, informed by the attachment framework, Kochanska et al. (2019) proposed that children’s positive representations of the parent as responsive and reliably providing comfort and effective help in times of stress can substantially defuse or buffer negative effects of parental power assertion. This hypothesis was supported in several studies (Herbert et al., 2026; Kochanska & An, 2024a).

We extend those findings to the current study to examine whether a similar moderation process can be identified in examining the effects of parental power assertion on children’s hostile mindset. We expected that children’s positive representations of the parent would moderate negative effects of power assertion and thus would account for multifinality, with the effects defused or buffered for children who view their parents as caring, trustworthy, and responsive, but present for children who view their parents as rejecting, unresponsive, and untrustworthy.

## The current study

We followed Dodge’s (Dodge, 2024; Dodge et al., 2022) recent model of a trait-like approach to hostile (or defensive) mindset that subsumes the consecutive social-information processing steps. To that effect, we aggregated across children’s responses to questions that targeted the consecutive steps and across several instruments utilizing vignettes depicting instances of harm/provocation and rejection in ambiguous peer contexts.

As mentioned earlier, we know relatively little about effects of parenting on hostile mindsets in low-risk families. In those families, parents routinely use power-assertive control to discipline and regulate behavior of their young children (Ritchie, 1999; Vittrup et al., 2006), but parental control rarely, if ever, rises to harmful, abusive, or threatening levels. Further, almost all studies have been conducted only with mothers and children, leaving a substantial gap in our knowledge about paternal influence. To address these limitations, we studied a community sample of generally well-functioning families with typically developing children (Children and Parents Study, CAPS), and collected parallel data in mother- and father–child dyads to permit the exploration of the examined processes in the two relationships.

We tested our comprehensive model of paths from early parental control to children’s future hostile mindset. The model encompasses both equifinality (mediation via both ToM and regulation) and multifinality (moderation by children’s representation of the parent). Utilizing observational and interview measures, we assessed parenting at toddler and preschool age, children’s ToM and regulation at preschool age, and children’s hostile mindsets and representations of the parent at early school age.

## Method

### Participants

CAPS included 200 families in U.S. Midwest (mothers, fathers, and typically developing 8-month-old biological children, 96 girls and 104 boys, born in 2017 and 2018). Parents responded to recruitment flyers, advertisements, posters, and social media posts distributed in various venues frequented by families. They were mostly White, but 20% of families included one or both non-White parents. Supplemental Table S1 presents demographic information.

All data were collected during 2–3.5-hour scripted sessions conducted by experimenters (Es) in a naturalistic university laboratory including a Living Room and a Play Room. We report data collected at age 3, *N* = 175, 86 girls, 89 boys, at age 4.5, *N* = 177, 86 girls, 91 boys, and at age 6.5 years, *N* = 153, 80 girls, 73 boys. Attrition was mostly due to the COVID-19 pandemic; some families chose to provide online data (included in the *N*s reported above). The sessions were videorecorded for future coding by multiple teams. Between 15% and 20% of cases were routinely used for reliability, followed by frequent realignments. Parents completed informed consent, and the Institutional Review Board at the University of Iowa approved the study (Children and Parents Study, CAPS, 201701705).

### Predictors: mothers’ and fathers’ power-assertive control, age 3–4.5

At age 3 years and at age 4.5 years, parental power assertion was coded in contexts designed to elicit typical control: toy cleanup (10 min) and naturalistic interactions, including introduction to the laboratory (5 min), snack prohibition (5 min), snack (5 min), and play (5 min). For details, see J. Kim and Kochanska (2026a) and S. Kim and Kochanska (2026). Details of coding and data reduction are in Supplement 2. Briefly, we coded parental power assertion for each 30-s segment in toy cleanups and for each 20-s segment in naturalistic interactions, using one of four codes that reflected increasing use of power assertion, from 1 = no control to 4 = forceful, harsh control (reliability, weighted kappas, 0.61–0.92 for toy cleanup and 0.83–0.85 for naturalistic interactions). To create robust scores based on 60 min of coded observations, we weighted and aggregated the scores across both contexts for age 3 and age 4.5, and across both ages, forming one composite power assertion score for each parent. Boys received more power assertion than girls from mothers, boys, *M* = 0.20, *SD* = 0.75, girls, *M* = -0.19, *SD* = 0.64, *t*(169) = -3.65, *p* < .001, and from fathers, boys, *M* = 0.17, *SD* = 0.74, girls, *M* = -0.19, *SD* = 0.68, *t*(159) = -3.21, *p* = .002.

### Mediator: children’s ToM, age 4.5

The description is brief, as details are in Kochanska et al. (2025). We used four well-established false-belief tasks (Hughes et al., 2005): unexpected contents (Band-Aid Box story), unexpected location (Andy’s Apple and New Toy stories), and belief-desire (Juice story). The tasks employed appropriate pictures, puppets, and props. Coding followed Hughes et al. (2005, pp. 369–370). Coders assigned scores of either 0 or 1 to each critical test question asked per story (the child had to respond correctly to control questions to earn a point). Reliability, kappas, ranged from 0.87 to 1.00. The total ToM score was the sum of scores across tasks. For analyses, we standardized this total score. There was no difference due to child gender.

### Mediator: children’s regulation, age 4.5

We used tasks calling for effortful control, or deliberate suppression of the dominant response and performing a subdominant response (Kochanska et al., 2000; Rothbart & Bates, 2006), in two delay tasks and one set-shifting task (see J. Kim & Kochanska, 2026a and Supplement 2, for details of procedures and coding). The two delay tasks were essentially similar, with slightly different details, and both involved the child waiting to open a gift. The set-shifting, Stroop-like task, Day/Night and Snow/Grass, was adapted from Carlson & Moses (2001).

Children’s performance correlated across the tasks (ranging from .18, *p* = .03 to .53, *p* < .001), with higher scores indicating better regulation, and they were aggregated into a composite of regulation. Girls outperformed boys, girls, *M* = 0.10, *SD* = 0.59, boys, *M* = -0.13, *SD* = 0.63, *t*(155) = 2.27, *p* = .012.

### Moderator: children’s representations of the parents, age 6.5

E presented the child with three stories depicting the child with the mother and three with the father. Small doll figures and props (furniture, dishes, trees, etc.) were used in all stories. The mother and father stories were analogous (Heavy pitcher/Hot pizza, Scooter/Teeter-Totter, Centipede/Rat, and a warmup story, Birthday Party, not coded). Originally inspired by MacArthur Story Stems Battery (MSSB; Bretherton et al., 1990; Buchsbaum & Emde, 1990; Holmberg et al., 2007; Oppenheim et al., 1997), the stories were further adapted from Davies et al. (2018). The parent issued a prohibition or a stern directive to the child (e.g., “Don”t touch hot food”; “Go to bed”), and the child disobeyed and was hurt (burned, injured), or the child became frightened. Having presented the story stem, E asked the child to show and tell what happened next. Details of stories and coding are in Supplement 2.

For each story, we coded Good Representation of the parent, i.e., children’s representations of the parent as responsive and reliably providing comfort and effective help in times of stress, operationalized as depiction of the parent as protective, forgiving, helpful, empathic, reassuring, trustworthy, and competent. We followed several studies that used similar story stems. Those studies defined “positive representation of the parent” as a portrayal of the story parent as protective, providing caregiving, caring, affectionate, warm, or helpful (Toth et al., 1997, 2000). We coded Good Representation from 0 = no evidence, to 1 = some evidence, to 2 = clear evidence, to 3 = strong, somewhat consistent, detailed evidence, to 4 = rich, abundant evidence. Reliability, weighted kappa, was .81. We also coded evidence of the story parent providing comfort (Comforting) as 0 = absent, 1 = present. Reliability, kappa, was .82.

The scores correlated across the three stories for both Good Representations, *rs* (139) = 0.39–0.65 and *rs* (123) = 0.36–0.49, *ps* < 0.001, and Comforting, *rs* (139) = 0.19–0.50 and *rs* (123) = 0.26–0.37, *ps* < 0.05, 0.01 or 0.001, for mother–child and father–child dyads, respectively. Thus, the scores were averaged across the three stories. The two scores correlated, *r*(139) = .67 and *r*(123) = .75, both *ps* < 0.001, for mother and father representations, respectively; they were standardized and aggregated into the score of the child’s positive representation (of each parent). Higher scores denote more positive representations. Girls had more positive representations of their mothers than boys, girls, *M* = 0.33, *SD* = 0.62, boys, *M* = -0.40, *SD* = 1.04, *t*(96.86) = 4.92, and of their fathers, girls, *M* = 0.26, *SD* = 0.72, boys, *M* = -0.31, *SD* = 1.06, *t*(94.06) = 3.38, both *ps* < 0.001.

### Outcome: children’s hostile mindset, age 6.5

#### Ambiguous stories

We adapted (with permission) eight stories, each accompanied by a color vignette, from the original battery (Dodge et al., 1990, 1995; Weiss et al., 1992), depicting a child protagonist and another child or children (all matching the participant in gender). The protagonist, to whom E refers as “you” (“imagine that you are …”), is a recipient of a harmful act (in six stories, physical harm, e.g., hit by a ball, tripped and falling, and in two stories, peer rejection, e.g., rebuffed when wanting to join peers in play). The pictures made the intent of the other child(ren) ambiguous by carefully depicting neutral facial expressions. E read each story to the child, and then, pointing to the protagonist, asked the child a series of five consecutive questions (“why do you think the other child [describe behavior]”, “did s/he do this on purpose or not”, “what would be the first thing you would feel”, ‘what would be the first thing you would want to do”, “what would be a good thing to do”).

***Coding*.** For each question, two aspects of the child’s response were coded: Hostile and benign (the latter not considered here). Hostile responses included attribution of intent (“because he wanted to hurt me”, “he hates me”, “on purpose”), feeling angry, wanting to yell, hit, kick, hurt him, and viewing an aggressive response as good. The codes ranged from 0 = no evidence, to 1 = unclear/maybe, or 2 = strong, clear evidence. Reliabilities of coding, weighted kappas, ranged from .65 to .94.

***Data reduction***. First, for each series of stories (six depicting harm, and two depicting rejection), we computed means for each hostile response across the stories (for each of the five questions). We then averaged those scores across the harm and rejection stories (all correlations were significant at *p* < .001). Finally, to form the overall composite score of hostile responses in the stories, we averaged those five variables (Cronbach’s alpha = 0.65).

#### Cartoons

This measure was based on Intention Attribution Test for Children (IAT; Vanwalleghem et al., 2020). We used a series of 12 (out of 16) cartoon strips, each consisting of three color vignettes, presenting situations in which the child protagonist experiences harm caused by another character whose intention is ambiguous or the act is nonintentional. We followed the procedure from Vanwalleghem et al. (2020): Upon the presentation of each strip, E asked the child to imagine that he or she was the protagonist and then asked what happened in the pictures. If the child’s response did not clearly indicate hostile or benign intention attributed to the harm doer, E asked standard follow-up questions, to the point of a forced choice. Responses to each cartoon strip were coded as 1 = hostile attribution, or 0 = non-hostile attribution. We used the sum of the former across the 12 cartoon strips as the overall composite of hostile responses.

#### Overall hostile mindset score

The two hostile response composites – for the stories and the cartoons – correlated, *r*(132) = .45, *p* < .001, and they were standardized and combined into an overall hostile mindset score. Boys’ scores were higher than girls, boys, *M* = 0.26, *SD* = 1.00, girls, *M* = -0.20, *SD* = 0.75, *t*(137) = -3.11, *p* = .002. All descriptive data are in Table 1.

**Table 1.** Descriptive data for all measures

Parallel measures for mother–child and father–child dyads										
Constructs	Mother–child dyads					Father–child dyads				*p*
	*M*		*SD*	*N*		*M*	*SD*		*N*	
Power-assertive control, age 3–4.5 years ^a^	0.01		0.72	171		−0.00	0.74		161	—
Power-assertive control, age 3 years ^a^	0.00		0.83	157		−0.00	0.86		149	—
Cleanup	42.48		6.24	157		43.80	7.09		147	.001
Naturalistic interactions	1.41		0.23	157		1.39	0.21		149	.001
Power-assertive control, age 4.5 years ^a^	0.00		0.84	156		0.00	0.77		147	—
Cleanup	41.76		5.49	156		41.46	6.27		147	.003
Naturalistic interactions	1.43		0.20	156		1.38	0.17		147	.001
Children’s positive representations of the parent, age 6.5 years ^a^	0.00		0.91	139		0.00	0.93		123	—
Good representation	2.50		0.79	139		2.53	0.75		123	.001
Comforting	0.76		0.32	139		0.79	0.27		123	.001

Child measures										
Constructs		*M*			*SD*			*N*		
Emotion dysregulation, age 8 months		0.00			1.00			197		
Theory of mind, age 4.5 years		2.19			2.14			157		
Regulation, age 4.5 years ^a^		−0.02			0.62			157		
Hostile mindset, age 6.5 years ^a^		0.01			0.90			139		
Stories		0.56			0.31			139		
Cartoons		4.37			3.21			132		

*Note*. ^a^ Composite of standardized constituent items, listed below. *P* refers to the difference between mother–child and father–child dyads, where applicable.

### Covariates

We covaried child gender and children’s emotion dysregulation at 8 months. Emotion dysregulation was assessed during the Car Seat episode from the Laboratory Temperament Assessment Battery (Goldsmith & Rothbart, 1999). The child was buckled tightly in a car seat for 60 s, and coders rated their overall emotion regulation based on the levels of distress and the number of emotion regulation strategies on a 5-point scale from 0 = unregulated (no control of distress) to 4 = well-regulated (completely regulates distress and distracts away from the source of distress), adapted from Perry et al. (2018). For analysis, we standardized and reversed scores to reflect emotion dysregulation. Reliability, intra-class correlations, ranged from 0.95 to 0.99.

## Results

### Preliminary analyses

Families that did and did not return at 6.5 years (and were seen in the laboratory) did not differ on any measures, except one: Children who returned had higher ToM scores than those who did not return, *M* = 2.35 vs. *M* = 1.33, *t*(37.34) = -2.52, *p* = .016. Further, among children who did not return, there were more boys (*N* = 41) than girls (*N* = 20); among the children who returned, there were fewer boys (*N* = 63) than girls (*N* = 76), χ^2^ = 8.14, *p* = .004. The data were considered missing completely at random (MCAR) according to Little’s (1988) MCAR test, χ^2^(15) = 15.32, *p* = .43 for mother–child dyads, χ^2^(25) = 27.36, *p* = .34 for father–child dyads.

Correlations are in Table 2. Several correlations were similar in mother- and father–child dyads: Children who received more power-assertive control were more poorly regulated and had higher hostile mindset scores. In mother–child dyads only, children who received more power-assertive control had lower ToM skills and less positive representations of the mothers.

**Table 2.** Correlations among study variables

Constructs	Children’s emotion dysregulation, 8 months	Mothers’ power-assertive control, age 3–4.5 years	Fathers’ power-assertive control, age 3–4.5 years	Children’s theory of mind, age 4.5 years	Children’s regulation, age 4.5 years	Children’s representations of the mother, age 6.5 years	Children’s representations of the father, age 6.5 years
Mothers’ power-assertive control, age 3–4.5 years	−.15						
Fathers’ power-assertive control, age 3–4.5 years	−.04	.56***	—				
Children’s theory of mind, age 4.5 years	−.03	−.26**	−.13	—			
Children’s regulation, age 4.5 years	.07	−.38***	−.31***	.29***	—		
Children’s representations of the mother, age 6.5 years	.13	−.18*	−.13	.05	.15	—	
Children’s representations of the father, age 6.5 years	.10	−.12	−.13	−.06	.13	.58***	
Children’s hostile mindset, age 6.5 years	−.07	.28**	.30***	−.31***	−.31***	−.10	−.09

*p < .05, **p < .01, ***p < .001.

The two mediators, ToM and regulation, were positively correlated, and both were associated with lower scores on hostile mindsets. The cross-parent correlations were significant for parental power assertion and children’s representations.

### Longitudinal links from parents’ power-assertive control to children’s hostile mindset: testing mediation via children’s ToM and regulation and moderation by children’s representations of the parent

We tested longitudinal mediation and moderation in Mplus 7 (Muthén & Muthén, 1998-2012) with 5,000-sample bootstrapping. We used a full information maximum likelihood estimator to handle missing data. Parental power-assertive control at age 3–4.5 was modeled as the predictor, children’s ToM and their regulation at age 4.5 as parallel mediators, allowed to covary, children’s hostile mindsets at age 6.5 as the outcome, and their positive representations of the parents at age 6.5 as the moderator of the relations between the predictor and the outcome. We covaried child gender and emotion dysregulation measured at 8 months. The significance of mediation was tested using a bias-corrected 95% confidence interval (CI). For significant moderation, we probed and plotted simple slopes at 1 *SD* above and below the mean of children’s positive representations of the parents (Aiken & West, 1991).

#### Mother-child dyads

**Figure 2.**
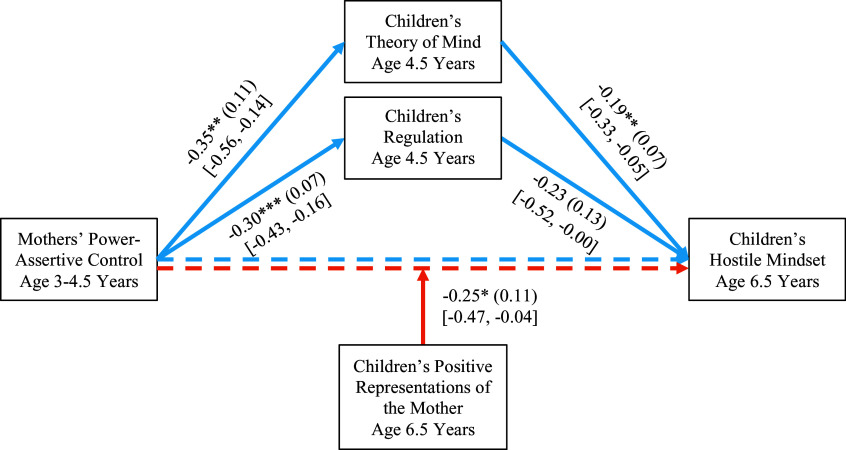
Longitudinal links from mothers’ power-assertive control to children’s hostile mindset mediated via children’s theory of mind and regulation and moderated by children’s representations of the mother. Unstandardized coefficients, standard errors in parentheses, and 95% confidence intervals in brackets are presented for significant paths. Children’s gender and emotion dysregulation at 8 months were covaried but are not depicted for clarity. *p < .05, **p < .01, ***p < .001. The relations between mothers’ power-assertive control and children’s hostile mindset were mediated via children’s theory of mind, *B* = 0.07, *SE* = 0.03, 95% CI [0.02, 0.15], and via regulation, *B* = 0.07, *SE* = 0.04, 95% CI [0.01, 0.18].

The results are in Figure 2. Children who had experienced more power-assertive control from mothers at age 3–4.5 had poorer ToM and regulation at age 4.5, which in turn led to more hostile mindsets at age 6.5. Although the path from regulation to hostile mindsets slightly missed significance, indirect effects were significant via both mediators, *B* = 0.07, *SE* = 0.03, 95% CI [0.02, 0.15] via ToM and *B* = 0.07, *SE* = 0.04, 95% CI [0.01, 0.18] via regulation.

**Figure 3.**
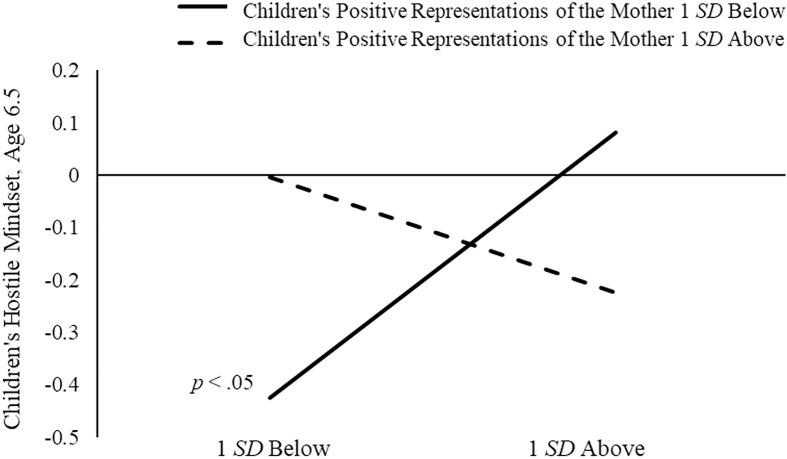
Children’s positive representations of the mother as moderators of the relations between mothers’ power-assertive control and children’s hostile mindset. Simple slopes were plotted holding children’s gender (girls = 0), early emotion dysregulation, theory of mind, and regulation constant.

We found significant moderation by children’s positive representations of the mother at age 6.5 (see Figure 3 for simple slopes). Children who had experienced more maternal power assertion had higher hostile mindset scores, but only if they had less positive representations of their mothers, *B* = 0.35, *SE* = 0.14, *p* < .05, 95% CI [0.08, 0.62]; there was no relation between maternal power assertion and children’s hostile mindsets for children whose representations of their mothers were positive. Thus, children’s positive representations of the mother (as accepting, responsive, trustworthy) buffered the negative effects of mothers’ higher power-assertive control on children’s hostile mindsets.

#### Father-child dyads

**Figure 4.**
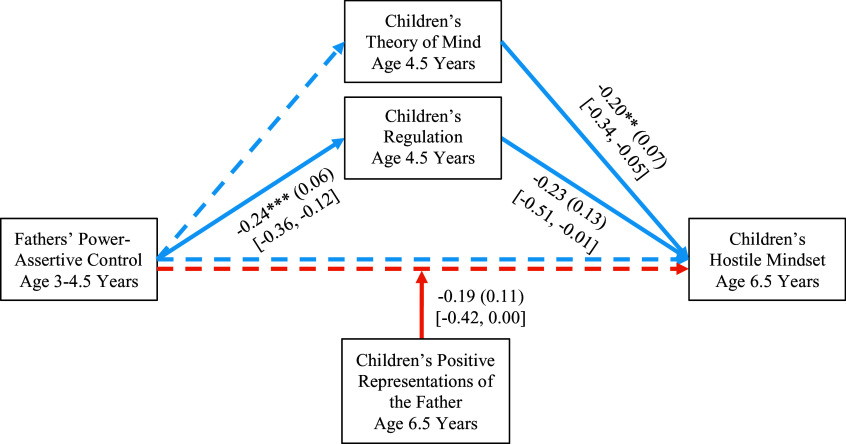
Longitudinal links from fathers’ power-assertive control to children’s hostile mindset mediated via children’s theory of mind and regulation and moderated by children’s representations of the father. Unstandardized coefficients, standard errors in parentheses, and 95% confidence intervals in brackets for significant paths are presented. Children’s gender and emotion dysregulation at 8 months were covaried but are not depicted for clarity. *p < .05, **p < .01, ***p < .001. The relations between fathers’ power-assertive control and children’s hostile mindset were mediated via children’s regulation, *B* = 0.05, *SE* = 0.03, 95% CI [0.01, 0.15].

The results are in Figure 4. We found similar patterns of results. Fathers’ higher power-assertive control at age 3–4.5 was related to lower levels of regulation in children at age 4.5, and lower levels of ToM and regulation at age 4 were associated with more hostile mindsets at age 6.5. However, the path from fathers’ power-assertive control at age 3.5–4 to children’s ToM at age 4.5 was not significant. Mediation was significant via children’s regulation, *B* = 0.05, *SE* = 0.03, 95% CI [0.01, 0.15]. Children who had experienced more paternal power assertion at age 3–4.5 had lower levels of regulation at age 4.5, which in turn led to more hostile mindsets at age 6.5.

**Figure 5.**
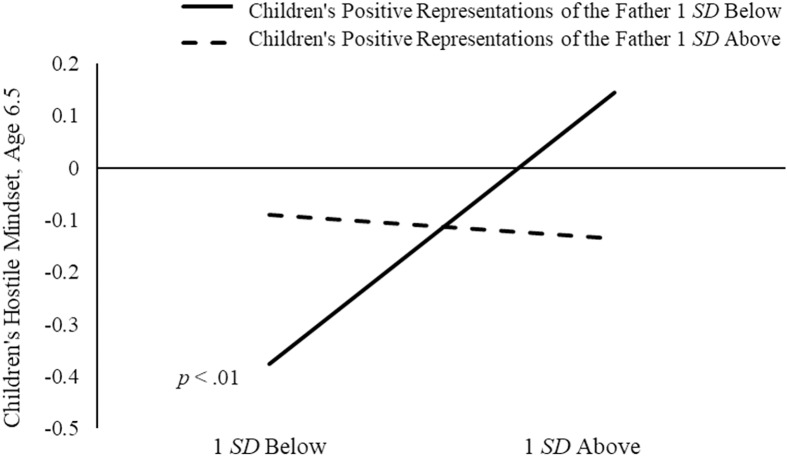
Children’s positive representations of the father as moderators of the relations between fathers’ power-assertive control and children’s hostile mindset. Simple slopes were plotted holding children’s gender (girls = 0), early emotion dysregulation, theory of mind, and regulation constant.

As in mother–child dyads, the effect of fathers’ power-assertive control on children’s hostile mindsets was moderated by children’s positive representations of the father (see Figure 5 for simple slopes). Higher paternal power-assertive control was related to more hostile mindsets only for children with less positive representations of their fathers, *B* = 0.35, *SE* = 0.10, *p* < .01, 95% CI [0.16, 0.56], but not for children with representations of their fathers as responsive and trustworthy, again supporting the buffering effect of children’s positive representations.

### Sensitivity analyses

Because we aggregated the power assertion scores (the predictor) across ages 3 and 4.5 to achieve a robust score based on 60 minutes of observations, we also examined whether the mediation results would be comparable for the disaggregated scores, i.e., for parental power assertion at a single time point. To that effect, we estimated two additional mediational models using parental power-assertive control measured at age 3 years only and at age 4.5 years only, with child gender and emotion dysregulation covaried. Across both these models, the patterns of mediation results were similar to the results from our original model with a composite power assertion score. The findings were as follows (the result for the model with the predictor at age 3 first, for the model with the predictor at age 4.5 next): For mother–child dyads, we found significant mediation via both ToM, *B* = 0.05, *SE* = 0.03, 95% CI [0.01, 0.12]; *B* = 0.05, *SE* = 0.03, 95% CI [0.01, 0.11], and regulation, *B* = 0.05, *SE* = 0.03, 95% CI [0.01, 0.14]; *B* = 0.08, *SE* = 0.04, 95% CI [0.02, 0.19]. For father–child dyads, we found significant mediation via regulation only, *B* = 0.04, *SE* = 0.03, 95% CI [0.004, 0.112]; *B* = 0.06, *SE* = 0.03, 95% CI [0.01, 0.15].

## Discussion

The model of children’s hostile information processing as a key antecedent of aggression and more broadly antisocial conduct problems, pioneered by Dodge and his colleagues, has long been one of the most influential conceptual frameworks in developmental psychology and psychopathology and beyond. Impressively, it has been supported by evidence collected across multiple cultures (Dodge et al., 2015).

Although generally hostile mindsets are rightly seen as maladaptive due to their proximal causal link to aggression, some caveat is warranted, and Dodge’s (2024) recent reconceptualization of them as “defensive” is instructive. In adverse, unstable, harsh, unpredictable, or violent early environments, being vigilant toward threats and prepared to retaliate may, in fact, be adaptive (Ellis & Del Giudice, 2019; Ellis et al., 2022; Pollak et al., 2005). Davies et al. (2019) demonstrated that in families characterized by caregivers’ intimate relationship instability, preschool children developed a dominant, coercive, and aggressive pattern of response, as assessed by typical vignettes targeting hostile mindsets. In those adverse circumstances, hostile mindsets may serve as unique cognitive protective factors. However, as the child begins to function in expanded social ecologies (peer groups, school), those mindsets undermine adaptive goals by interfering with prosocial, cooperative, socially skillful, or academically successful behavior, and lead instead to social rejection, school failure, and delinquency (Dodge et al., 2003; Lansford et al., 2010).

Scientific success of any theory is measured not only by its large impact and robust empirical support, but also by its heuristic potential. Both are certainly true of Dodge and colleagues’ research on children’s hostile social-information processing: Although impact and supporting evidence keep accumulating, new compelling issues that inspire more research questions also arise. Toward that goal, we asked two questions that explore further the well-established association between power-assertive parenting and the formation of children’s hostile mindset: Can we elucidate specific mechanisms that account for that association, and can we elucidate factors that moderate it?

The first question is one of equifinality in development. We proposed that parental power assertion promotes children’s hostile mindset via two paths or mediators – the child’s diminished abilities to understand accurately others’ perspectives (i.e., ToM) and to regulate their own behavior. The literature has supported links between parenting and each of these mediators and between them and children’s hostile mindsets, but to our knowledge, no study has formally examined the parallel mediation in a longitudinal design.

The second question is one of multifinality in development. Here, we proposed that children’s representations of the parent as either caring, responsive, and reliably providing comfort and help, or rejecting, unresponsive, and unavailable in times of distress and stress can significantly buffer or amplify the effects of power-assertive parenting, even in a sample of families in which power assertion was quite mild.

Our findings largely supported our expectations and were mostly replicated across mother- and father–child relationships. We found evidence of equifinality: As hypothesized, the association between power-assertive parental control and children’s hostile mindset was mediated by children’s ToM skills (for mothers and children) and by children’s regulation skills (for both relationships).

It is unclear why we failed to detect the presence of an indirect effect of fathers’ power assertion on children’s hostile mindset via ToM; note that paternal power assertion, in contrast to maternal, was unrelated to children’s ToM. Empirical evidence on differences in links between various aspects of parenting and children’s ToM across mother- and father–child dyads is sparse and far from consistent (Pavarini et al., 2013). Goffin et al. (2020) found a significant longitudinal association between mothers’ mind-mindedness in infancy and children’s ToM at preschool age but failed to find a parallel effect for fathers. However, Kochanska et al. (2025), drawing from two studies, reported that in both samples, there were longitudinal associations between father–child mutually responsive orientation and child ToM; but for mothers and children, that link was found in only one of the two samples. Those reports, however, did not include data on power assertion.

It is important to keep in mind that the concept of power assertion is difficult to conceptualize and measure, as it is deeply embedded in cultural contexts (Deater-Deckard et al., 2011; Lansford et al., 2022, 2024). Parents’ views on discipline strategies that benefit children, on circumstances that merit the use of parental pressure or power, and their choices of physical, emotional, and verbal control techniques vary depending on the rules and norms prevalent in a given culture, on parental socioeconomic and educational status, and racial and ethnic background. As reviewed earlier, physical discipline techniques may be considered accepted, normative, and benefitting the child in some cultures, and disapproved and even legally prohibited in others. The complexity may be reflected also in children’s interpretations of parental power assertion and control more generally. In cultures where such strategies are normative, children may perceive them as acceptable. In other cultures, for example, relatively educated U. S. samples like ours, children may view even a sharp rebuke or a light slap as harsh, hostile, and mean spirited. This led us to consider children’s representations of the parent as key to understanding multifinality in effects of power assertion.

We indeed found evidence of multifinality, and those findings were replicated across mother- and father–child dyads. As hypothesized, children’s representations of the parent as caring, responsive, and reliably helpful at times of stress buffered the negative effects of power-assertive parenting at toddler and preschool ages on hostile mindsets at early school age. Importantly, those findings align with previous studies that had demonstrated moderating effects of positive relational experiences – and further, specifically experiences assessed at the level of representation – on the relations between parents’ power assertion and children’s various socialization outcomes (Bendel-Stenzel et al., 2023; Herbert et al., 2026; Kochanska & S. Kim, 2012; Kochanska & An, 2024a; Kochanska et al., 2019). Further, at the broader conceptual level, those findings are synergistic with the recently growing emphasis on the significance of children’s subjective construals of their experiences rather than of “objective” parameters of those experiences (Smith & Pollak, 2021; L. A. Sroufe & J. Sroufe, 2025). It appears that the child’s perception or representation of the parent as reliably available, competent, and supportive was an important factor determining whether parental power-assertive control would contribute to the child’s future hostile mindset or worldview.

As a caveat, it is possible that children’s positive representations of parents can only buffer detrimental effects of parental power assertion in families that use mild levels of power, as has been the case in this and earlier studies reporting similar findings (Herbert et al., 2026; Kochanska & An, 2024a). However, Toth et al. (2002), studying maltreated children, likely to have experienced severe harsh discipline, reported similar significant negative correlations between children’s positive perceptions of their mothers and child externalizing problems. Alink et al. (2009) reported similar findings, with children’s positive representations of their mothers buffering negative effect of maltreatment on child emotion regulation. Clearly, more research on when, how, and for whom children’s representations of parents serve as significant moderators in socialization processes is needed.

Another important consideration in interpreting the current findings is the possibility of child effects on parenting. Long-standing research has demonstrated that child factors such as difficult temperament and regulatory capacities can shape parental responses and behaviors (e.g., Bell, 1968; Klein et al., 2018; J. Kim & Kochanska, 2026b; Sanson et al., 2004). To partially address this concern, we controlled for the effects of children’s emotion dysregulation in infancy, but other child-related factors, including genetic influences, may also contribute to parental power assertion (Shewark et al., 2021). Admittedly, in future work, more robust measures of “child effects” would be desirable, for example, multi-method assessments of anger proneness or difficult temperament. Those may elucidate more complex dynamics of child characteristics potentially influencing future parenting, as well as children’s regulatory capacities, and finally, also their tendency to develop hostile mindsets.

As well, biological and genetic factors can contribute to the emergence and intergenerational transmission of hostile mindsets. Dodge (2009) proposed an elaborate model, in which “… in response to possible threat, three systems coactivate: Low monoamine oxidase A (MAOA) activity is associated with a pattern of autonomic arousal and defensive information-processing that is characterized by hypervigilance to hostile cues, hostile attributional biases, selection of self-defensive goals, and the experience of self-righteous anger” (p. 411). Galán et al. (2017) empirically demonstrated that MAOA and harsh parenting assessed in early childhood interact to predict children’s hostile social information processing.

Further, parents and children may share genetic disposition toward hostile mindsets. We assessed parents’ hostility using the Aggression Questionnaire (Buss & Perry, 1992), but we found no significant associations between those scores and children’s hostile mindsets. However, future research measuring parents’ and children’s characteristics, including genetically mediated traits, is warranted to further disentangle bidirectional influences between parents and children.

The strengths of this work include all observational measures, parallel data from mother- and father–child dyads, and a longitudinal design, with the outcome assessed at early school age, when hostile mindset is especially important, because it triggers the long-lasting and “snowballing” negative cascades (Dodge et al., 2003; Lansford et al., 2010). The nature of the sample was a limitation, and may limit generalizability to other cultural contexts, as described earlier. Our participants came from community families, relatively homogenous ethnically (although recall that 20% of families were not “White alone” and the sample was well representative of the state from which it was drawn). Children were typically developing, by and large well adjusted; parents used mostly mild and reasonable power assertion tactics when controlling their children’s behavior. Note, however, that the typical and low-risk nature of our sample might be an advantage, given that most studies of hostile mindsets have involved children with elevated levels of aggression.

However, our model did assume that generally, hostile mindsets are not adaptive, given the relatively benign social environments in which our participating children lives. In future research, it will be important to replicate our equifinality-multifinality model of hostile mindsets in families living in difficult circumstances, dealing with challenges such as intimate partner violence, lack of stability, or a high-crime neighborhood. In such harsh circumstances, the processes we outlined may work differently: As mentioned earlier, a hostile or defensive mindset can be adaptive, and consequently, a child who is proficient in reading others’ mental states would be correct in interpreting others’ intentions as hostile and evaluating aggressive responses as desirable.

Like any empirical study of a complex developmental process, this work leaves many unexplored questions. As indicated at the outset, we limited our review to parental power assertion, but other parenting aspects are certainly linked with children’s ToM and emotion regulation, and thus they may also play a role in the unfolding hostile mindsets. Aspects of positive parent–child relationships, such as security, positive mutuality, mutual responsiveness, and especially various forms of parental positive mentalization, have been associated with better ToM skills (e.g., Aubuchon et al., 2023; Hughes et al., 2005; Kochanska et al., 2025; Licata, 2016; Malcorps et al., 2023; McCormick et al., 2022; Meins et al., 2002; Pavarini et al., 2013; Szpak & Bialecka-Pikul, 2020) and with better regulation (e.g., Boldt et al., 2020; Samdan et al., 2020) and would consequently be expected to be linked to children’s less hostile mindsets and more benign worldviews. Many exciting directions of research remain to be explored.

## Supporting information

10.1017/S0954579426101333.sm001 Kochanska et al. supplementary material 1 Kochanska et al. supplementary material

10.1017/S0954579426101333.sm002 Kochanska et al. supplementary material 2 Kochanska et al. supplementary material

## Data Availability

Data are not available. Analysis code is available on request. Methods are available on request. We are unable to share data for individual families. Our consent forms the parents signed clearly preclude any sharing of individual data, even if deidentified. The parents have consented to the data being shared in the aggregate form only, and we are ethically and legally bound to follow this agreement.

## Supplementary material

The supplementary material for this article can be found at https://doi.org/10.1017/S0954579426101333.

## Pre-registration statement

This study was not pre-registered. Although we had not pre-registered the current hypotheses and models, we have previously presented relevant findings from our research program in multiple empirical and theoretical papers (many of them cited in the current paper). Additionally, we specified our model in our grant applications, and that information is publicly available.
