# Supplementary material for: Equifinality and multifinality in developmental paths to a hostile mindset: A longitudinal study of mother–child and father–child dyads from toddler to early school age
Source: Dev Psychopathol. 2026 Mar 27:1–14. Online ahead of print. doi: 10.1017/S0954579426101333 (PMC13038403; doi:10.1017/S0954579426101333)
Supplement: Kochanska et al. supplementary material 1 — Kochanska et al. supplementary material [file S0954579426101333sup001.docx]

Multifinality and equifinality in developmental paths to a hostile mindset: A longitudinal study of mother-child and father-child dyads from toddler to early school age.

Supplement 2: Coding and Data Aggregation for Selected Constructs

(Contact the authors for complete coding manuals)

**Mothers’ and Fathers’ Power-assertive Control, Age 3-4.5**

We coded parental power assertion for each 30-s segment in toy cleanup and for each 20-s segment in naturalistic interactions, using the following codes: *No Control* (no interaction, purely social exchange, play), *Gentle Guidance* (gentle, subtle, playful suggestion or direction), *Control* (non-forceful, matter-of-fact, relatively assertive control), and *Forceful, Harsh Control* (negative, power-assertive control often accompanied with hostility, frustration, or threats). The verbal, affective, and physical markers of each code were extensively defined.

We tallied the instances of each code, created relative scores by dividing each tally by the number of coded segments (for naturalistic interactions only; hence the different metrics for the final scores in cleanup vs. interactions), weighted each score by multiplying *No Control* by 1, *Gentle Guidance* by 2, *Control* by 3, and *Forceful or Harsh Control* by 4, and added and standardized them. The scores for toy cleanup and naturalistic interactions correlated, at age 3, mothers, *r*(157) = .38, *p* < .001, and fathers, *r*(147) = .48, *p* < .001, and at age 4.5, mothers, *r*(156) = .42, *p* < .001, and fathers, *r*(147) = .19, *p* = .022. Therefore, at each age, we created a single power assertion score for each parent by averaging them, with higher scores indicating higher power assertion.

The power assertion scores correlated robustly across both assessments, *r*(142) = .50 and *r*(135) = .50, *p*s < .001, for mothers and fathers, respectively. Consequently, to create robust scores based on 60 min of coded observations, we aggregated the scores for age 3 and age 4.5 for each parent.

**Children’s Regulation, Age 4.5**

***Gift-Delay – Wrap and Bow***

This paradigm was conducted in mother-child sessions. Experimenter (E) brought in a loosely wrapped gift and asked the child not to peek while he or she wrapped the rest of the gift. E wrapped the gift noisily for 60 s while turned away from the child. Child behavior was coded as 0 = *turns around, doesn’t return fully forward*, 1 = *turns around but turns back forward,* 2 = *peeks over shoulder far enough to see the wrapping,* 3 = *turns head to side but less than 90 degrees,* or 4 = *does not try to peek.* Latency to the violation of the instruction was coded in seconds (60 if the child never tried to peek). After 60 s, E stated that he or she had forgotten the bow and asked the child to stay in the chair and not touch or peek the gift until he or she returns. E left the child alone in the room for 3 min. Child behavior was coded as 0 = *opens gift,* 1 = *lifts up gift*, 2 = *touches gift but does not lift*, 3 = *does not touch gift.* Child seat score was coded as 0 = *in seat for less than 30 s,* t 1 = *in seat for more than 30 s but less than 1 min*, 2 = *in seat for more than 1 min but less than 2 min,* or 3 = *in seat for more than 2 min.* Latencies to violation and to leaving the seat were coded in seconds (180 if never). A single score was created by standardizing and averaging the behavior scores, seat scores, and latency.

***Gift Delay – Bag***

This paradigm was conducted in father-child sessions. E brought in a gift in a bag and stated that he or she had forgotten a bow. E asked the child to stay in their chair and not peek in the bag until he or she had returned. E left the child alone in the room with the bag for 3 min. Child behavior was coded as 0 = *removes gift from bag,* 1 = *puts hand into bag but does not remove gift*, 2 = *peeks in, with or without touching,* 3 = *touches bag without peeking,* or 4 = *does not touch bag or peak.* Child seat score and latencies to the violation and leaving the seat were coded identically to those in Gift-Delay – Wrap and Bow. A single score was created by standardizing and averaging the behavior score, seat score, and latencies.

***Day/Night and Snow/Grass***

This paradigm was conducted in father-child sessions. E showed the child two cards, one depicting a nighttime sky and the other a daytime sky and asked them to pick the nighttime sky when E said “Day” and the daytime sky when E says “Night” for 10 trials (5 each for Day and Night). The procedure was repeated for Snow/Grass. Child responses were coded from 0 = *fails to point,* to 1 = *incorrect and never self-corrects,* to 2 = *self-corrects,* to 3 = *correct on first attempt and doesn’t change mind.* Scores were summed and standardized.

**Children’s Representations of the Parents, Age 6.5**

During each session (one per parent), each child viewed four narratives. The first story was a warm-up story and was not coded; three narratives per parent were coded. Each story was analogous across parents (Heavy pitcher/Hot pizza; Scooter/Teeter-Totter; Centipede/Rat). In each story, the parent issued a prohibition or instruction, and the child disobeyed and was either hurt or became frightened.

Shortly after E introduced the main stem of the story (see below for story prompts), E asked the child to complete, develop, or contribute details to the story (“Show me what happens next”) with props, including a parent doll and child doll. Children were encouraged to handle the dolls to promote engagement. Nondirective comments such as “Does anything else happen in the story?”, “What does Dad say or do?”, and “What do you think Mom is thinking?” were used, but questions guiding or directing the story were not. When the child addressed the main issue in the story, E waited for a natural pause and then asked, “How does the story end?” Afterwards, E moved onto the next story.

***Story Stems***

**Juice Pitcher Story Stem (Mother).** In this story stem, the mother makes the child juice in a pitcher. The mother leaves the room to grab some cups but tells the child not to drink from the pitcher because it is too heavy. When the mother leaves the room, the child tries to drink out of the pitcher but drops it on their foot and spills the juice on the floor.

**Scooter Story Stem (Mother).** In this story stem, the mother and child are outside in the yard. The child asks the mother to ride the scooter, but the mother says she needs to go and get the helmet and knee pads first. The mother leaves to grab them but tells the child not to ride the scooter until she returns because it isn’t safe. The child rides the scooter, then falls and hurts themselves.

**Centipede in Sink Stem (Mother).** In this story stem, the mother tells the child to go brush their teeth before bed. The child thinks they see a centipede in the sink and becomes fearful. The child goes to the mother and tells her that they are scared.

**Hot Pizza Stem (Father).** In this story stem, the father makes the child pizza. The father says that the pizza is very hot, and that the child needs to wait until it cools down to eat it, then leaves the room. The child then takes a bite of the pizza but burns their tongue and drops the pizza on the floor.

**Teeter-Totter Stem (Father).** In this story stem, the father and child are at the park. The child starts to walk on the teeter-totter, but the father tells them not to because they could lose their balance and fall. The child walks on the teeter-totter and falls and hurts their arm.

**Rat in Bedroom Stem (Father).** In this story stem, the father tells the child to go to sleep. The child goes to their room but thinks they see a rat under the bed and becomes fearful. The child then runs to their father and tells him they are scared.

***Coding***

**Good Representation of Parent.** In response to child distress, parent may be depicted as**protective, affectionate, forgiving,**and **helpful.** Parent may also be depicted as **warm, composed,**and **emotionally present.** Parent may seem to see, understand, and respond to how child feels, or otherwise be characterized as **empathic**or **reassuring.**Additionally, parent may be**resourceful, trustworthy,** and**knowledgeable;** that is, parent finds solutions (e.g., provides a bandage or chases away/gets rid of the rat). Another important consideration is if parent’s response successfully promotes child composure or return to play. Theoretically, this presumably indicates that the child's representation of the parent is positive, and that the child may perceive the parent to be a consistent, reliable, trustworthy, and effectual source of comfort and knowledge.

Consider frequency of occurrence and intensity when assigning overall code for each story for Good Representation of Parent. Also, consider when and how the child introduces parent into story. Note that a higher score may be earned by frequent instances or fewer but intense instances.

**0 –** **no evidence.**

**1 –** **some evidence present**. This evidence is incomplete, ambiguous, fleeting, or trivial.

**2 – clear evidence present.**Child may show one instance of parent being affectionate, resourceful, or helpful (i.e., picks up the pitcher); however, no pattern emerges – this could feel disjointed from the rest of the narrative, or the rest of the narrative may be sparse.

**3 – strong, somewhat consistent, detailed evidence present.**Child mentions multiple instances of parent being affectionate, resourceful, or helpful.

**4 – rich, abundant, and exceptionally thorough evidence present.**Child may require little prompting, and the story is in portraying the parent as having a consistent and warm presence in the narrative, or multiple details indicate a significant role in alleviating child distress. This story may seem cohesive.

**Parent Comfort**. Code for depiction of parent as a source of comfort. This can include parent as a source of affection, reassurance, or protection (e.g., removes the centipede). Do not code for instrumental help, or help that could be classified as ‘housekeeping,’ such as cleaning up spilled lemonade.

**0 – absent**

**1 – present**
