# Supplementary material for: Equifinality and multifinality in developmental paths to a hostile mindset: A longitudinal study of mother–child and father–child dyads from toddler to early school age
Source: Dev Psychopathol. 2026 Mar 27:1–14. Online ahead of print. doi: 10.1017/S0954579426101333 (PMC13038403; doi:10.1017/S0954579426101333)
Supplement: Kochanska et al. supplementary material 2 — Kochanska et al. supplementary material [file S0954579426101333sup002.docx]

Equifinality and multifinality in developmental paths to a hostile mindset: A longitudinal study of mother-child and father-child dyads from toddler to early school age.

**Table S1**

***Demographic characteristics of the recruited sample at entry (N = 200)***

| Characteristic | *M* or % | *SD* |
| --- | --- | --- |
| Child gender | 48% girls |  |
| Child age at entry (months) | 8.05 | 0.78 |
| Family annual income |  |  |
| Less than $10,000 | 2.0% |  |
| $10,001 – $20,000 | 3.5% |  |
| $20,001 – $30,000 | 4.0% |  |
| $30,001 – $40,000 | 5.5% |  |
| $40,001 – $50,000 | 7.5% |  |
| $50,001 – $60,000 | 8.0% |  |
| $60,001 – $70,000 | 8.0% |  |
| $70,001 – $80,000 | 8.5% |  |
| $80,001 – $90,000 | 7.0% |  |
| $90,001 – $100,000 | 13.5% |  |
| $100,001 – $150,000 | 27.0% |  |
| More than $150,001 | 5.5% |  |

Family structure

Married 87%

Not married but living together 11.5%

Other 1.5%

| Mothers | |  | Fathers | |
| --- | --- | --- | --- | --- |
| Characteristic | *M* or % | *SD* | *M* or % | *SD* |
| Age (years) | 31.07 | 4.42 | 32.94 | 5.65 |
| Education |  |  |  |  |
| Did not complete high school | 0% |  | 1.0% |  |
| High school | 14.5% |  | 23.0% |  |
| Associate degree | 11.5% |  | 6.5% |  |
| Bachelor’s degree | 35.0% |  | 37.0% |  |
| Advanced degree | 39.0% |  | 32.5% |  |
| Ethnicity |  |  |  |  |
| Hispanic or Latino | 4.5% |  | 1.5% |  |
| Not Hispanic or Latino | 95% |  | 98.5% |  |
| Unknown | 0.5% |  | 0% |  |
| Race |  |  |  |  |
| Asian | 5.5% |  | 3.5% |  |
| Black or African American | 1.5% |  | 3.0% |  |
| White | 88.5% |  | 88.5% |  |
| More than one race | 4.5% |  | 3.5% |  |
| Unknown | 0% |  | 1.5% |  |
